# Characteristics of patients with SARS-COV-2 PCR re-positivity after recovering from COVID-19

**DOI:** 10.1017/S0950268823000249

**Published:** 2023-02-17

**Authors:** Cheng-Yi Hu, Yi Lei, Yu-Wen Tang, Wen-Shuai Cui, Pei-Lian Wu, Yan-Fang Li, Yan Zhou, Xin-Yan Li, Hao Cui, Lu-Shan Xiao, Zhu-Xiang Zhao

**Affiliations:** 1 Department of Infectious Diseases, Guangzhou First People's Hospital, School of Medicine, South China University of Technology, Guangzhou, Guangdong, China; 2 Department of Respiratory Disease, Eighth People's Hospital of Guangzhou, Guangzhou Medical University, Guangzhou, China; 3 Department of Infectious Diseases, Nanfang Hospital, Southern Medical University, Guangzhou, China

**Keywords:** Clinical characteristics, COVID-19, predictive model, repeat test positivity, SARS-CoV-2 infection

## Abstract

The purpose of this study was to analyse the clinical characteristics of patients with severe acute respiratory syndrome coronavirus 2 (SARS-COV-2) PCR re-positivity after recovering from coronavirus disease 2019 (COVID-19). Patients (*n* = 1391) from Guangzhou, China, who had recovered from COVID-19 were recruited between 7 September 2021 and 11 March 2022. Data on epidemiology, symptoms, laboratory test results and treatment were analysed. In this study, 42.7% of recovered patients had re-positive result. Most re-positive patients were asymptomatic, did not have severe comorbidities, and were not contagious. The re-positivity rate was 39%, 46%, 11% and 25% in patients who had received inactivated, mRNA, adenovirus vector and recombinant subunit vaccines, respectively. Seven independent risk factors for testing re-positive were identified, and a predictive model was constructed using these variables. The predictors of re-positivity were COVID-19 vaccination status, previous SARs-CoV-12 infection prior to the most recent episode, renal function, SARS-CoV-2 IgG and IgM antibody levels and white blood cell count. The predictive model could benefit the control of the spread of COVID-19.

## Introduction

More than 600 million cases of coronavirus disease 2019 (COVID-19), including more than 6.5 million deaths, were reported to the World Health Organization up to 7 September 2022 [1]. Despite many previous studies regarding the clinical features of COVID-19 [2–4], limited information is available on the clinical characteristics of patients who have recovered from severe acute respiratory syndrome coronavirus 2 (SARS-CoV-2) infection.

Recently, positive SARS-CoV-2 reverse-transcription polymerase chain reaction (RT-PCR) results have been reported after hospital discharge in some patients who have recovered from COVID-19 [5, 6]. However, other studies have not found re-positive results in recovered patients during the long-term follow-up [7, 8]. Opinions differ regarding whether re-positivity is due to re-infection or a false-positive result or delayed release of the virus. It is not yet known whether re-positive patients are contagious and should be isolated.

A previous meta-analysis found that 12.2% patients who had been discharged from hospital retested positive during the convalescent period [9]. However, the re-positivity rate reported in different studies is variable. In Italy, about 50% of patients who recovered from COVID-19 were still positive for SARS-CoV-2 RNA on retesting 3 weeks after the onset [10]. However, in China only 12.2% of patients have positive SARS-CoV-2 PCR test results after discharge from hospital [11]. The proportion of recovered COVID-19 patients with re-positive SARS-CoV-2 PCR results requires clarification. The time frame for retesting positive was not completely consistent in different studies (Supplementary Table S1), but most patients who tested re-positive for SARS-CoV-2 RNA had re-positive results within 30 days of discharge. The difference in the time frame might account for the difference in the rate of re-positive.

The risk factors for re-positivity are unclear. A study of the clinical characteristics of 59 patients with SARS-COV-2 PCR re-positivity after recovering from COVID-19, found that age, sex, severity of disease and time from onset to hospitalisation were not significantly associated with re-positivity [12]. Breakthrough SARS-CoV-2 infections have been observed globally in both vaccinated and previously infected individuals [13, 14]. Re-positive results have been reported in recovered patients infected with the Omicron variant [15]. However, it is unclear whether the incidence of re-positivity is associated with the infecting SARS-CoV-2 variant.

In addition, it is important to find out whether re-positive results are associated with complications in recovered patients, and whether re-positive patients need to be followed up. An analysis of data on 93 patients with re-positive results found that most patients were asymptomatic and that a minority had mild symptoms [16]. However, another study found a case fatality rate of 0.4% in patients with re-positive results [11].

The aim of this study was to determine the proportion of recovered COVID-19 patients with re-positive results, the clinical characteristics of these patients and the effects of re-positivity on recovered patients and to develop a predictive model to identify patients who are more likely to have re-positive results.

## Methods

### Patient cohort and study design

A retrospective cohort study was conducted of patients admitted to Guangzhou First People's Hospital with COVID-19 between 7 September 2021 and 11 March 2022 who had recovered and been discharged. All patients were admitted to the Eighth People's Hospital of Guangzhou for treatment within 48 h of confirmed SARS-CoV-2 infection. After the patients met government requirements for recovery from COVID-19, they were transferred to the Guangzhou First People's Hospital for isolation, monitoring and rehabilitation training. The duration of this post-recovery quarantine and rehabilitation period was stipulated by the government as at least 2 weeks, and these patients underwent routine SARS-CoV-2 PCR testing at least twice a week during the quarantine period. A nucleic acid detection kit based on the fluorescent PCR method was used to detect ORF1ab and N fragments in samples collected by throat swab. In the analysis, patients were divided into two groups according to whether they retested positive (re-positive) on SARS-CoV-2 PCR testing during their post-recovery stay in Guangzhou First People's Hospital.

### Definitions

Several criteria were required for discharge from the Eighth People's Hospital of Guangzhou and Guangzhou First People's Hospital [5]: (1) normal temperature lasting more than 3 days, (2) resolved respiratory symptoms, (3) substantially improved acute exudative lesions on chest computed tomography (CT) and (4) two consecutive negative SARS-CoV-2 RT-PCR test results at least 24 h apart.

Some of the participants in the study had been re-infected with SARS-CoV-2. SARS-CoV-2 re-infection was defined as a clinical recurrence of COVID-19 symptoms, accompanied by a positive SARS-CoV-2 PCR test result more than 3 months after the original diagnosis [17].

In the study, patients were classified as ‘re-positive’ only if both the following conditions were met: (1) the patient had two consecutive negative SARS-CoV-2 PCR test results at least 24 h apart before being discharged from the Eighth People's Hospital of Guangzhou; and (2) the patient had at least one positive SARS-CoV-2 PCR test result within 2 weeks during the rehabilitation period in the Guangzhou First People's Hospital.

The estimated glomerular filtration rate (eGFR) was calculated using the Modification of Diet in Renal Disease equation.

### Data collection

Clinical electronic medical records, laboratory test results and radiological reports of all patients who had recovered from COVID-19 were reviewed. Detailed data, including demographic information, epidemiological information, comorbidities, signs and symptoms, laboratory test results, imaging reports, treatment and outcomes, from Guangzhou First People's Hospital were collected for each patient. In addition, patients' laboratory test results and imaging reports within 3 days of confirmed SARS-CoV-2 infection were extracted from the admission records of Guangzhou First People's Hospital. The end point of this study was discharge from Guangzhou First People's Hospital. A trained team of physicians and researchers collaborated to crosscheck the patient data and verify the data accuracy.

### Statistical analyses

SPSS Version 25.0 (IBM, Armonk, New York, USA) software was used for statistical analyses. As Shapiro–Wilk normality test showed that none of the continuous variables in this study were normally distributed, continuous variables were reported as medians with interquartile ranges. The categorical variables were reported as frequencies and percentages. The test-positive and test-negative groups were compared using the Mann–Whitney *U* test for continuous variables and the chi-squared test or Fisher's exact test for categorical variables. Univariable and multivariable logistic regression models were used to explore the risk factors associated with re-positivity. In the univariable logistic regression analyses, variables with *P* < 0.05 were regarded as potential risk factors and were included in the multivariable logistic regression analysis using a forward elimination procedure (likelihood ratio test and elimination if *P* > 0.1). Receiver-operating characteristic curve (ROC) analyses were performed to assess the discrimination and calibration of the model. The areas under the ROC curve (AUC) were compared using DeLong's Test. The cut-off value of the predictive model score was selected based on the maximum value of Youden's index. In addition, the chi-squared test was also used to analyse the impact of the type and number of vaccinations and the effect of SARS-CoV-2 infection prior to the start of the study on re-positive results. All statistical tests were two-sided, and *P*-values less than 0.05 were regarded as statistically significant.

## Results

### Description of the study population

**Fig. 1.**
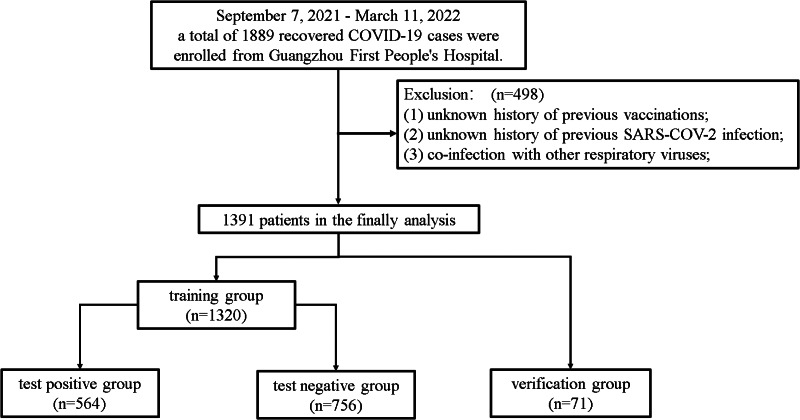
Flowchart of patient selection.

Data were collected on a total of 1889 patients admitted to the Guangzhou First People's Hospital between 7 September 2021 and 11 March 2022 after recovering from COVID-19. A total of 498 patients with an unknown previous vaccination history, a SARS-COV-2 infection history or co-infection with other respiratory viruses, were excluded, leaving 1391 patients in the analysis. Of the 1391 patients, 1320 patients were assigned to the training group, and 71 patients were assigned to the validation group. Among the patients in the training group, 564 (42.7%) had a re-positive SARS-CoV-2 PCR test result during their stay in the Guangzhou First People's Hospital. These 564 patients were assigned to the ‘test-positive group’, and the other 756 patients were assigned to the ‘test-negative group’. A flow chart of study participant selection is shown in Figure 1. None of the patients with re-positive test results were observed to be infectious.

### Patient characteristics according to re-positive status during the post-COVID-19 quarantine and rehabilitation period

Compared with the patients in the test-negative group (Table 1), fewer patients in the test-positive group had received 2–4 doses of COVID-19 vaccine (75.9% *vs.* 81.9%, *P* = 0.008) or had had two separate episodes of COVID-19 (8.2% *vs.* 13.0%, *P* = 0.006). Compared with patients in the test-negative group, patients in the test-positive group had lower SARS-CoV-2 immunoglobulin G (IgG) levels (6.987 [interquartile ranges: 6.849–7.920] *vs.* 7.753 [6.931–8.088]; *P* < 0.001), and higher SARS-CoV-2 immunoglobulin M (IgM) levels (1.920 [0.427–24.986] *vs.* 0.992 [0.255–5.510]; *P* < 0.001). Additionally, patients in the test-positive group were more likely to have an eGFR ≥ 90 ml/min/1.73 m^2^ (77.8% *vs.* 69.4%, *P* = 0.001).

**Table 1.** Demographics and clinical characteristics of the patients during the post-COVID-19 quarantine and rehabilitation period

	Training group (*n* = 1320)	Test positive group (*n* = 564)	Test negative group (*n* = 756)	*P*-value
Age (year)	35 (28–46)	35 (28–45)	36 (29–46)	0.085
Sex (man)	1031 (78.1%)	428 (75.9%)	603 (79.8%)	0.092
The doses of the COVID-19 vaccine				0.008
0–1	273 (20.7%)	136 (24.1%)	137 (18.1%)	
2–4	1047 (79.3%)	428 (75.9%)	619 (81.9%)	
Number of episodes of COVID-19				0.006
1	1176 (89.1%)	518 (91.8%)	658 (87.0%)	
2	144 (10.9%)	46 (8.2%)	98 (13.0%)	
Comorbidities				
Hypertension	111 (8.4%)	50 (8.9%)	61 (8.1%)	0.606
Diabetes	41 (3.1%)	24 (4.3%)	17 (2.2%)	0.038
Coronary heart disease	7 (0.5%)	4 (0.7%)	3 (0.4%)	0.469
Chronic hepatitis	74 (5.6%)	32 (5.7%)	42 (5.6%)	0.926
Tumour history	7 (0.5%)	3 (0.5%)	4 (0.5%)	>0.999
Hyperlipidaemia	174 (13.2%)	67 (11.9%)	107 (14.2%)	0.227
Hyperuricemia	278 (21.1%)	112 (19.9%)	166 (22.0%)	0.355
Sleep disorder	8 (0.6%)	6 (1.1%)	2 (0.3%)	0.080
Anxiety or depression	13 (1.0%)	7 (1.2%)	6 (0.8%)	0.415
Symptoms and signs				
Cough	15 (1.1%)	10 (1.8%)	5 (0.7%)	0.059
Expectoration	8 (0.6%)	4 (0.7%)	4 (0.5%)	0.730
Fever	2 (0.2%)	0 (0)	2 (0.3%)	0.510
Shortness of breath	2 (0.2%)	2 (0.4%)	0 (0)	0.182
Chest tightness	3 (0.2%)	1 (0.2%)	2 (0.3%)	>0.999
Fatigue	1 (<0.1%)	0 (0)	1 (0.1%)	>0.999
Laboratory findings				
C-reactive protein (mg/l)	0.80 (0.35–1.74)	0.80 (0.36–1.75)	0.83 (0.34–1.78)	0.704
White blood cell (10^9/l)	6.22 (5.26–7.37)	6.14 (5.06–7.19)	6.29 (5.33–7.50)	0.030
Lymphocyte ratio (%)	34.8 (30.1–39.5)	35.3 (30.3–40.3)	34.3 (29.9–39.0)	0.031
Neutrophil counts (10^9/l)				0.033
<2	94 (7.1%)	50 (8.9%)	44 (5.8%)	
≥2	1226 (92.9%)	514 (91.1%)	712 (94.2%)	
eGFR (ml/(min × 1.73 m^2^))				0.001
<90	356 (27.0%)	125 (22.2%)	231 (30.6%)	
≥90	964 (73.0%)	439 (77.8%)	525 (69.4%)	
Urea nitrogen (mmol/l)	4.33 (3.69–4.98)	4.27 (3.64–4.91)	4.38 (3.73–5.02)	0.630
Aspartate transaminase (U/l)	21 (18–28)	22 (17–28)	21 (18–28)	0.933
Alanine transaminase (U/l)	25 (15–41)	24 (14–40)	25 (15–41)	0.486
Total bilirubin (μmol/l)	10.8 (8.6–13.3)	10.9 (8.7–13.2)	10.8 (8.5–13.5)	0.955
Direct bilirubin (μmol/l)	2.0 (1.5–2.5)	2.0 (1.5–2.5)	2.0 (1.5–2.6)	0.307
Albumin (g/l)				0.367
<35	21 (1.6%)	11 (2.0%)	10 (1.3%)	
≥35	1299 (98.4%)	553 (98%)	746 (98.7%)	
Creatine kinase (U/l)	63 (49–83)	63 (47–84)	63 (50–82)	0.633
Creatine kinase isoenzymes (U/l)	11 (9–14)	11 (9–14)	11 (8–14)	0.663
SARS-CoV-2 IgG (S/CO)	7.50 (6.85–8.09)	6.99 (6.85–7.92)	7.75 (6.93–8.09)	<0.001
SARS-CoV-2 IgM (S/CO)	1.22 (0.31–5.80)	1.92 (0.43–24.99)	0.99 (0.26–5.51)	<0.001
Chest CT image				
Without lung inflammation	459 (34.8%)	201 (35.6%)	258 (34.1%)	0.568
With inflammatory nodules	668 (50.6%)	268 (47.5%)	400 (52.9%)	0.053
With ground glass shadow	253 (19.2%)	122 (21.6%)	131 (17.3%)	0.049
With lung consolidation	17 (1.3%)	8 (1.4%)	9 (1.2%)	0.716
Progression^a^	237 (18.0%)	105 (18.6%)	132 (17.5%)	0.588
Treatment				
Use of antibiotics	10 (0.8%)	6 (1.1%)	4 (0.5%)	0.341
Use of antiviral drugs	7 (0.5%)	4 (0.7%)	3 (0.4%)	0.469
Using hormones	2 (0.2%)	0 (0)	2 (0.3%)	0.510

eGFR, estimated glomerular filtration rate

a Progression: the first chest CT result during quarantine observation and rehabilitation training was worse than the chest CT result when discharge from the Eighth People's Hospital.

Laboratory test and imaging results are based on the results of the first blood test and the first chest CT results during the quarantine and rehabilitation period.

### Differences in the clinical characteristics of the test-positive and test-negative groups at the time of their initial COVID-19 diagnosis

Compared with the patients in the test-negative group (Table 2), patients in the test-positive group had lower white blood cell (WBC) counts (6.19 [4.94–7.66] *vs.* 6.82 [5.49–8.04], *P* < 0.001) and higher SARS-CoV-2 IgG levels (211.47 [75.37–211.48] *vs.* 23.63 [23.62–122.72], *P* < 0.001), and lower SARS-CoV-2 IgM (3.68 [0.80–3.69] *vs.* 4.50 [0.463–4.50], *P* < 0.001).

**Table 2.** Laboratory test results within 3 days of confirmation of SARS-CoV-2 infection according to group

Laboratory findings	Training group (*n* = 1320)	Test positive group (*n* = 564)	Test negative group (*n* = 756)	*P*-value
White blood cell (10^9/l)	6.55 (5.29–7.93)	6.19 (4.94–7.66)	6.82 (5.49–8.04)	<0.001
Lymphocyte ratio (%)	32.6 (24.8–38.9)	32.6 (25.0–39.9)	32.4 (24.8–38.3)	0.314
Neutrophil counts (10^9/l)				<0.001
<2	90 (6.8%)	56 (9.9%)	34 (4.5%)	
≥2	1230 (93.2%)	508 (90.1%)	722 (95.5%)	
eGRR (ml/(min × 1.73 m^2^))				0.001
<90	552 (41.8%)	207 (36.7%)	345 (45.6%)	
≥90	768 (58.2%)	357 (63.3%)	411 (54.3%)	
Aspartate transaminase (U/l)	19 (15–24)	19 (15–24)	19 (16–24)	0.643
Total bilirubin (μmol/l)				0.165
>17.1	157 (11.9%)	59 (10.5%)	98 (13.0)	
≤17.1	1163 (88.1%)	505 (89.5%)	658 (87.0%)	
Total bilirubin (μmol/l)				0.385
>6.8	101 (7.7%)	39 (6.9%)	62 (8.2%)	
≤6.8	1219 (92.3%)	525 (93.1%)	694 (91.8%)	
Albumin (g/l)				0.411
<35	6 (0.5)	4 (0.7%)	2 (0.3%)	
≥35	1314 (99.5%)	560 (99.3%)	754 (99.7%)	
Creatine kinase (U/l)	86 (66–115)	77 (62–117)	91 (68–114)	<0.001
Creatine kinase isoenzymes (U/l)	11 (8–13)	9 (8–13)	12 (9–13)	<0.001
SARS-CoV-2 IgG (S/CO)	83.63 (23.63–211.47)	211.47 (75.37–211.48)	23.63 (23.62–122.72)	<0.001
SARS-CoV-2 IgM (S/CO)	3.69 (0.54–4.50)	3.68 (0.80–3.69)	4.50 (0.46–4.50)	<0.001

eGFR, estimated glomerular filtration rate

### COVID-19 vaccination status

The most widely used type of vaccine was inactivated vaccine, followed by mRNA, adenovirus vector and recombinant subunit vaccines (Supplementary Fig. S1). Of the study patients, 85%, 79%, 14%,and 0.2% had received one, two, three and four doses of vaccine, respectively. The number of patients vaccinated with different vaccines from the first dose to the fourth dose is shown in Table 3.

**Table 3.** Vaccination status in the training group

Number of vaccinated patients	Inactivated vaccines	mRNA vaccines	Adenovirus vector vaccines	Recombinant subunit vaccines	Not recorded	All
First dose	810	170	69	4	64	1117
Second dose	776	164	55	4	48	1047
Third dose	108	49	8	4	18	187
Forth dose	1	1	1	0	0	3

### Timing of re-positive SARS-CoV-2 PCR test results during the quarantine period

**Fig. 2.**
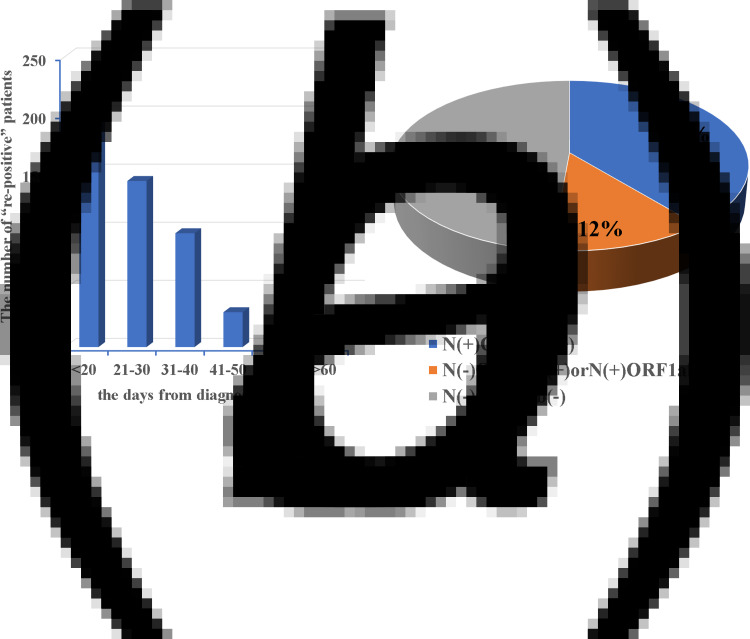
(a) Number of new SARS-CoV-2 patients with re-positive result according to the number of days since COVID-19 diagnosis in the training group; (b) results of the first SARS-CoV-2 RNA test during quarantine among re-positive patients recovering from COVID-19 in the training group.

Most patients who tested re-positive for SARS-CoV-2 RNA tested re-positive within 40 days of diagnosis (Fig. 2a). Among patients who tested re-positive, only 39% had both ORF1ab and N fragments detected on the first SARS-CoV-2 PCR test during the quarantine rehabilitation period (Fig. 2b).

### Effect of the Omicron variant on the proportion of patients with re-positive result

**Fig. 3.**
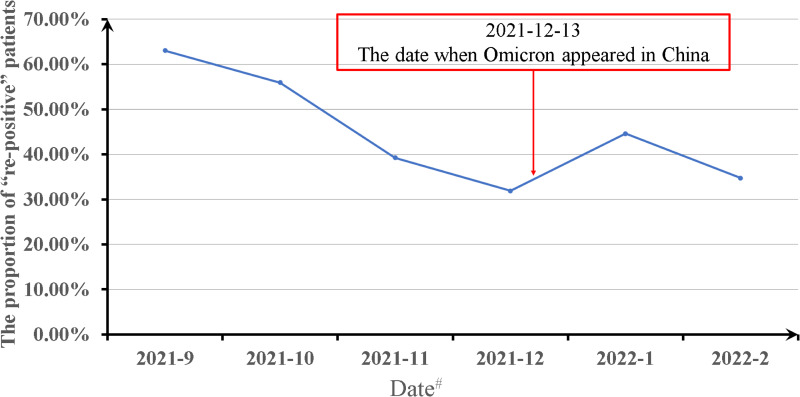
Effect of the Omicron variant on the incidence of re-positivity among patients recuperating from COVID-19 in the training group. Date#: the date when patients were assessed to have recovered and were discharged from the Eighth People's Hospital of Guangzhou.

After the Omicron variant was discovered in China, there was a transient increase in the proportion of patients with a re-positive result, followed by a gradual decline (Fig. 3).

### Development of the model to predict patients with a re-positive result

To effectively identify patients with a re-positive result, a clinical characteristic-based predictive model was developed. The multivariable logistic regression analysis (Table 4) identified the number of doses of COVID-19 vaccine, number of episodes of COVID-19, eGFR and SARS-CoV-2 IgM measured during quarantine; and WBC count SARS-CoV-2 IgG, and SARS-CoV-2 IgM at the time of diagnosis, as independent risk factors for re-positivity. The predictive model was constructed using these seven variables according to the following equation:

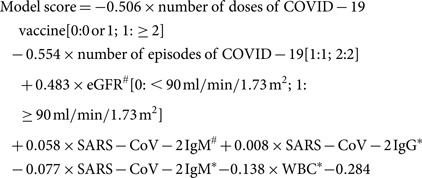

where ^#^ is the result during the post-COVID-19 quarantine and rehabilitation period, and * is the result at the time of diagnosis

**Table 4.** Results of the univariable and multivariable logistic regression analyses of risk factors for SARS-CoV-2 re-positivity in training group

	Univariate logistic regression		Multivariable logistic regression	
	*P*	OR	Adjust-*P*	OR
The doses of the COVID-19 vaccine (0–1 *vs.* 2–4)	0.008	0.697 (0.533–0.910)	0.002	0.603 (0.441–0.825)
Number of episodes of COVID-19 (1 *vs.* 2)	0.006	0.596 (0.412–0.862)	0.009	0.575 (0.379–0.872)
Diabetes	0.038	1.932 (1.028–3.632)		
During quarantine observation and rehabilitation training				
White blood cell (10^9/l)	0.030	0.921 (0.860–0.986)		
Lymphocyte ratio (%)	0.031	1.015 (1.002–1.029)		
Neutrophil counts (10^9/l) (<2 *vs.* ≥2)	0.033	1.574 (1.034–2.397)		
eGRR (ml/(min × 1.73 m^2^)) (<90 *vs.* ≥90)	0.001	1.545 (1.201–1.988)	0.001	1.622 (1.218–2.158)
SARS-CoV-2 IgG (S/CO)	<0.001	0.949 (0.894–1.007)		
SARS-CoV-2 IgM (S/CO)	<0.001	1.058 (1.043–1.074)	<0.001	1.060 (1.042–1.078)
With ground glass shadow	0.049	1.317 (1.000–1.734)		
At the time of diagnosis				
White blood cell (10^9/l)	<0.001	0.870 (0.822–0.922)	<0.001	0.871 (0.817–0.929)
Neutrophil counts (10^9/l) (<2 *vs.* ≥2)	<0.001	2.341 (1.506–3.638)		
eGRR (ml/(min × 1.73 m^2^)) (<90 *vs.* ≥90)	0.001	0.691 (0.553–0.863)		
Creatine kinase (U/l)	<0.001	1.000 (0.999–1.000)		
Creatine kinase isoenzymes (U/l)	<0.001	0.973 (0.965–0.982)		
SARS-CoV-2 IgG (S/CO)	<0.001	1.002 (1.001–1.002)	<0.001	1.008 (1.007–1.009)
SARS-CoV-2 IgM (S/CO)	<0.001	0.941 (0.917–0.966)	<0.001	0.926 (0.899–0.954)

eGFR, estimated glomerular filtration rate

**Fig. 4.**
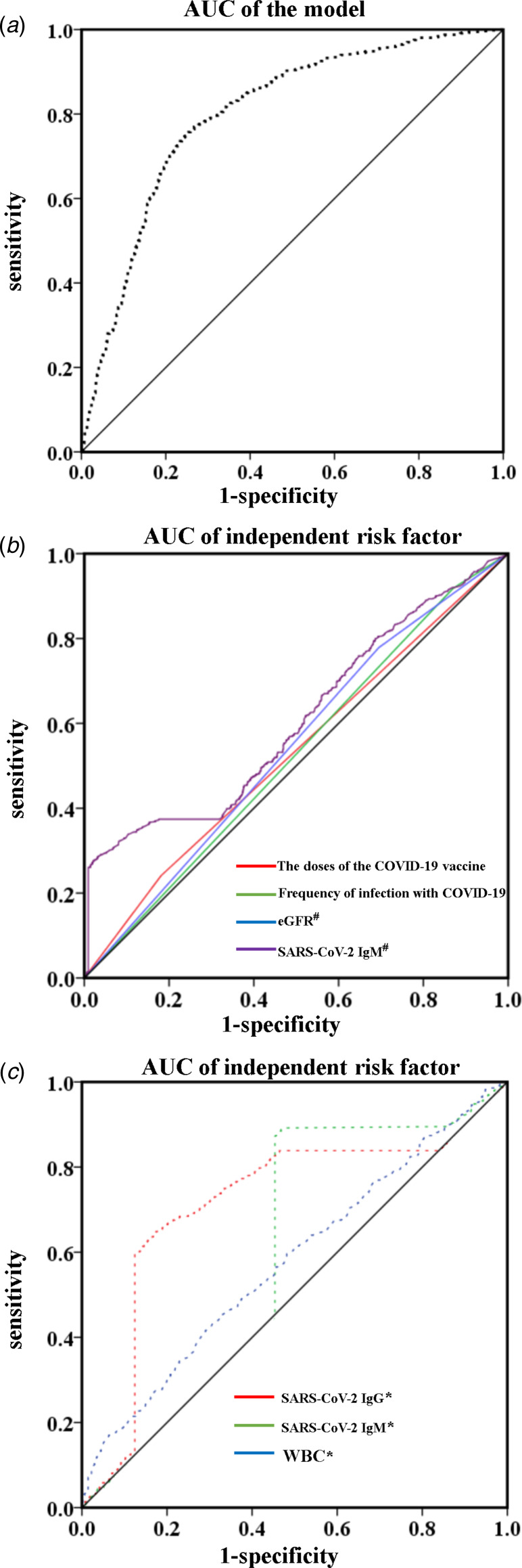
Model for the prediction of SARS-CoV-2 PCR re-positivity in patients who had recovered from COVID-19 in the training group. (a) Area under the curve (AUC) results of the model; (b and c) results for each independent risk factor. # Results during the post-COVID-19 quarantine and rehabilitation period. * Results at the time of diagnosis.

The model performed well for categorising patients into test-negative and test-positive groups (AUC: 0.800, 95% confidence interval: 0.776–0.824; *P* < 0.001) (Fig. 4a), and the model's ability to predict SARS-CoV-2 re-positivity was statistically significant (*P* < 0.001, Omnibus Tests of Model Coefficients). Based on the largest Youden's index (0.503), the optimal cut-off for stratifying patients into high-risk and low-risk test-positive groups was 0.411. The sensitivity and specificity of the model were 60.1% and 83.6%, respectively. Compared with each independent risk factor within the model (Figs 4b and 4c), the model score had the highest AUC (*P* < 0.001, Supplementary Tables S2 and S3), indicating that the model had higher predictive ability than any of the individual risk factors.

### Model evaluation

In order to verify the accuracy of the model, 71 patients (Supplementary Table S4) who recovered from COVID-19 were included in the study as the verification group, including 31 patients with a re-positive result. The sensitivity and specificity of the model in the verification group were 77.4% and 82.5%, respectively. The AUC in the verification group was 0.827 (95% confidence interval: 0.718–0.906; *P* < 0.001) (Supplementary Fig. S2).

### Effect of the dose and type of COVID-19 vaccine on re-positive status

**Fig. 5.**
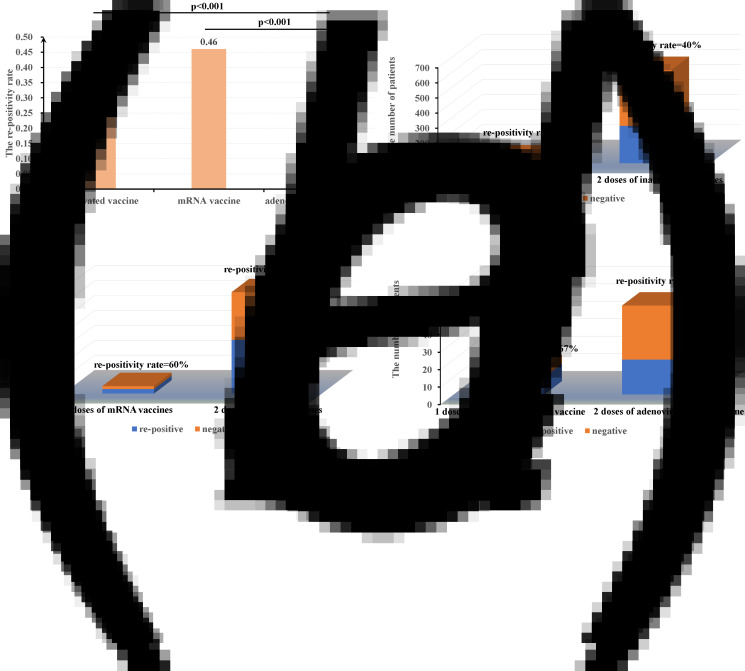
Frequency of testing re-positive in the training group according to the number of doses of COVID-19 vaccine received, and the vaccine type. (a) incidence of re-positive results according to COVID-19 vaccine type; incidence of re-positivity among patients who received only (b) inactivated vaccines, (c) mRNA vaccines or (d) adenovirus vector vaccines.

The re-positivity rate was 39%, 46% and 11% in patients who had received inactivated, mRNA and adenovirus vector vaccines, respectively (Fig. 5a). As only four patients received recombinant subunit vaccine, we did not calculate the re-positivity rate in these patients. Compared with patients who had received an adenovirus vector vaccine, the risk of testing re-positive was significantly higher in those who had received an inactivated vaccine or mRNA vaccine (both *P* < 0.001). However, the re-positivity rate was not significantly different between patients who had received inactivated and mRNA vaccines (39% *vs.* 46%, *P* = 0.081). Compared with unvaccinated patients, the risk of testing re-positive was significantly lower in those who had received an inactivated vaccine (39% *vs.* 50%, *P* = 0.007) or adenovirus vector vaccine (11% *vs.* 50%, *P* < 0.001). Among patients who only received inactivated vaccines or mRNA or adenovirus vector vaccines, the probability of testing re-positive showed a downward trend as the number of doses of COVID-19 vaccine increased although the results were not statistically significant (Figs 5b–d).

## Discussion

Considering that most patients with re-positive results were asymptomatic, it was hard to determine whether the re-positive results were due to re-infection or due to intermittent shedding of RNA fragments [18]. However, considering that these patients with re-positive result were not observed to be infectious during the rehabilitation period in the Guangzhou First People's Hospital, it is likely that a re-positive result was primarily due to intermittent shedding of RNA fragments, and not due to active infection. Although no evidence was found that the patients who tested re-positive were infectious [11, 19], the re-positive results showed these patients still had fragments of the virus in their bodies. Therefore, appropriate follow-up is necessary for recovered patients. In addition, false-negative SARS-CoV-2 PCR test results on discharge from hospital are also a factor that might lead to recovered patients having a re-positive result. Several potential factors may be responsible for false-negative SARS-CoV-2 PCR test results using respiratory samples on discharge from hospital [20], including the SARS-CoV-2 test kit, the sources of samples, sample transportation and the sampling procedure; thus, the addition of testing of faecal samples may be a good option for patients with COVID-19 who will be discharged immediately [21].

In our study, 43% recovered patients tested re-positive for SARS-CoV-2 RNA on PCR testing during the quarantine and rehabilitation period. Due to the large proportion of patients with a re-positive result, we recommend that the frequency of SARS-CoV-2 PCR testing of patients with COVID-19 should be increased before discharge to reduce the re-positivity rate after discharge [22]. Similar to our results, the re-positivity rate in the study by Cento *et al*. [10] was 46.9%. However, some studies have reported lower re-positivity rates of 3–25% [23–25]. Possible reasons for the variable re-positivity rates in different studies include: (1) some discharged patients in some studies did not undergo nucleic acid testing because they were asymptomatic; (2) the sample size in some studies was small and the patients were not representative; (3) variable timing of retesting after discharge; and (4) the frequency of weekly retesting was too low, resulting in missed diagnosis of some re-positive patients. In our study, 61.7% patients with a re-positive result received their first re-positive result within 30 days of diagnosis. We speculate that with the prolongation of the timing of retesting, the cumulative number of re-positive patients would gradually increase, because some recovered patients without re-positive result at the beginning would eventually have re-positive result in subsequent re-tests. In a study by Deng *et al*. [26], 77% of re-positive patients had positive results within 14 days of starting the quarantine and rehabilitation period, and the remaining 23% of re-positive patients had positive results after 14 days.

Through univariable analysis and multivariable logistic regression, we determined that the predictors of re-positivity were COVID-19 vaccination status, previous SARs-CoV-12 infection prior to the most recent episode, renal function, SARS-CoV-2 IgG and IgM antibody levels and WBC count. Increasing number of doses of COVID-19 vaccine and repeated episodes of COVID-19 enable memory B and T cells to activate the body's immunity more rapidly and potently, and SARS-CoV-2 is eliminated faster; thus the number of doses of COVID-19 vaccine and episodes of COVID-19 were negatively correlated with re-positivity. High levels of SARS-CoV-2 IgM in patients during quarantine and rehabilitation implied that the immune response had not subsided, and that the patient was excreting viral fragments, leading to the detection of re-positive results; thus, SARS-CoV-2 IgM in patients during quarantine and rehabilitation was positively correlated with re-positivity. Some previous studies have found that interleukin 6, lung images, age, sore throat, nasopharyngeal viral load and inefficient viral clearance were predictors of re-positivity [10, 23, 26–28]. However, no correlation was found between age and re-positivity in our study. In addition, 18% of patients in our study had worsening lung images, but progression of chest CT images was not a risk factor for re-positivity in our study. In addition, due to limitations of the retrospective study design, interleukin 6, sore throat, nasopharyngeal viral load and inefficient viral clearance were not included in the analysis.

Patients who tested re-positive after recovering from COVID-19 rarely had symptoms, such as fever, cough and expectoration and their WBC counts were relatively normal, consistent with the results of previous studies [9, 29]. In addition, liver function and myocardial enzymes of patients in the test-positive group did not differ significantly from those in the test-negative group, which indicates that re-positive status was not associated with damaged liver function or myocardial injury. Therefore, we believe that re-positivity does not lead to serious complications. Even though re-positive patients were asymptomatic in most cases and both re-positive patients and patients with asymptomatic infections have positive nucleic acid test results, re-positivity and asymptomatic infection are different. Re-positive patients are not contagious and are unlikely to develop complications, but re-infected patients are contagious and may develop serious complications.

Our study has several limitations. First, some clinical indicators were not included due to data unavailability or a large amount of missing data, which might be one of the reasons why the sensitivity and specificity of our model were not very high. Second, due to the resource limitations of SARS-CoV-2 test kits, patients were not tested for SARS-CoV-2 RNA daily during the post-COVID-19 quarantine and rehabilitation period, which led to a slight discrepancy between the actual date when the patients had re-positive results and the date we recorded. Finally, most of the patients in our study were Chinese, and there were only a few European, American and African patients. Therefore, it was not possible to assess the impact of racial or ethnic differences on the results of our study. These limitations might limit the reliability and generalisability of our model's results.

In conclusion, we identified the clinical characteristics of re-positive patients, which we hope will benefit the control of the spread of COVID-19.

## Data Availability

The data supporting the results of this study are available from the corresponding author on reasonable request.

## Supplementary material

For supplementary material accompanying this paper visit https://doi.org/10.1017/S0950268823000249. click here to view supplementary material
