# Supplementary material for: Characteristics of patients with SARS-COV-2 PCR re-positivity after recovering from COVID-19
Source: Epidemiol Infect. 2023 Feb 17;151:e34. doi: 10.1017/S0950268823000249 (PMC10019929; doi:10.1017/S0950268823000249)
Supplement: Supplementary file 1 [file S0950268823000249sup001.docx]

# *Epidemiology and Infection*

# Characteristics of patients with SARS-COV-2 PCR re-positivity after recovering from COVID-19

Cheng-Yi Hu, Yi Lei, Yu-Wen Tang, Wen-Shuai Cui, Pei-Lian Wu, Yan-Fang Li, Yan Zhou, Xin-Yan Li, Hao Cui, Lu-Shan Xiao, Zhu-Xiang Zhao

Supplementary Materials

**Supplementary Table S1.** The time frame for retesting positive for the cited studies

| Research | Time interval from discharge to test re-positive | Time interval from diagnosis to test re-positive | Detection frequency |
| --- | --- | --- | --- |
| **Lan L, et al.[1]** | 5 to 13 days | not mentioned | Three times in 4-5 days |
| **Liotti FM,** **et al.[2]** | not mentioned | about 48 to 57 days | not mentioned |
| **Ren X, et al.[3]** | most were within 30 days | not mentioned | not mentioned |
| **Cento V, et al.[4]** | 90.5% of cases were within one month | not mentioned | not mentioned |
| **Wu X, et al.[5]** | 1 to 165 days | not mentioned | once per month |
| **Shui TJ, et al.[6]** | 6 to 8 days | 23 to 27 days | not mentioned |
| **Li T, et al.[7]** | about within 28 days | not mentioned | not mentioned |
| **Yang C, et al.[8]** | about 8 days | not mentioned | 1 to 3 times per week |
| **Wu J, et al.[9]** | 3 to 15 days | not mentioned | once per 3 days |
| **Zou Y, et al.[10]** | 1 to 12 days | not mentioned | not mentioned |
| **Chen J, et al.[11]** | 3 to 18 days | 16 to 64 days | not mentioned |
| **Mei Q, et al.[12]** | 4 to 38 days | not mentioned | not mentioned |
| **Wong J, et al.[13]** | 11 to 18 days | not mentioned | not mentioned |
| **Deng W, et al.[14]** | most were within 14 days | not mentioned | not mentioned |
| **Zheng J, et al.[15]** | 5 to 8 days | not mentioned | once per 2-3 days |
| **Zheng KI, et al.[16]** | within 7 days | not mentioned | not mentioned |

**References**

1. **Lan L, et al.** (2020) Positive RT-PCR Test Results in Patients Recovered From COVID-19. *JAMA* **323**(15), 1502-1503.

2. **Liotti FM, et al.** (2021) Assessment of SARS-CoV-2 RNA Test Results Among Patients Who Recovered From COVID-19 With Prior Negative Results. *JAMA Intern Med* **181**(5), 702-704.

3. **Ren X, et al.** (2021) A systematic review and meta-analysis of discharged COVID-19 patients retesting positive for RT-PCR. *EClinicalMedicine* **34**, 100839.

4. **Cento V, et al.** (2020) Persistent positivity and fluctuations of SARS-CoV-2 RNA in clinically-recovered COVID-19 patients. *The Journal of infection* **81**(3), e90-e92.

5. **Wu X, et al.** (2021) A follow-up study shows that recovered patients with re-positive PCR test in Wuhan may not be infectious. *BMC Med* **19**(1), 77.

6. **Shui TJ, et al.** (2020) Characteristics of recovered COVID-19 patients with recurrent positive RT-PCR findings in Wuhan, China: a retrospective study. *BMC Infect Dis* **20**(1), 749.

7. **Li T, et al.** (2022) Clinical characteristics and risks of the convalescent COVID-19 patients with re-detectable positive RNA test: a 430 patients with Omicron infected cross-sectional survey in Tianjin, China. *J Infect Public Health* **15**(12), 1409-1414.

8. **Yang C, et al.** (2020) Viral RNA level, serum antibody responses, and transmission risk in recovered COVID-19 patients with recurrent positive SARS-CoV-2 RNA test results: a population-based observational cohort study. *Emerg Microbes Infect* **9**(1), 2368-2378.

9. **Wu J, et al.** (2020) Clinical characteristics and outcomes of discharged COVID-19 patients with reoccurrence of SARS-CoV-2 RNA. *Future Virol* **15**(10), 663-671.

10. **Zou Y, et al.** (2020) The Issue of Recurrently Positive Patients Who Recovered From COVID-19 According to the Current Discharge Criteria: Investigation of Patients from Multiple Medical Institutions in Wuhan, China. *J Infect Dis* **222**(11), 1784-1788.

11. **Chen J, et al.** (2020) Clinical course and risk factors for recurrence of positive SARS-CoV-2 RNA: a retrospective cohort study from Wuhan, China. *Aging (Albany NY)* **12**(17), 16675-16689.

12. **Mei Q, et al.** (2020) Assessment of patients who tested positive for COVID-19 after recovery. *Lancet Infect Dis* **20**(9), 1004-1005.

13. **Wong J, et al.** (2020) Probable causes and risk factors for positive SARS-CoV-2 test in recovered patients: Evidence from Brunei Darussalam. *J Med Virol* **92**(11), 2847-2851.

14. **Deng W, et al.** (2020) Positive results for patients with COVID-19 discharged form hospital in Chongqing, China. *BMC Infect Dis* **20**(1), 429.

15. **Zheng J, et al.** (2020) Incidence, clinical course and risk factor for recurrent PCR positivity in discharged COVID-19 patients in Guangzhou, China: A prospective cohort study. *PLoS Negl Trop Dis* **14**(8), e0008648.

16. **Zheng KI, et al.** (2020) A Case Series of Recurrent Viral RNA Positivity in Recovered COVID-19 Chinese Patients. *J Gen Intern Med* **35**(7), 2205-2206.

**Supplementary Table S2.** Area under the curve of the model and independent risk factors for SARS-CoV-2 re-positivity

| independent risk factor | AUC | p |
| --- | --- | --- |
| The model | 0.800(0.776-0.824) | p<0.001 |
| the doses of the COVID-19 vaccine | 0.530(0.498-0.562) | 0.062 |
| cumulative frequency of infection with COVID-19 | 0.524(0.493-0.555) | 0.135 |
| eGFR^#^ | 0.542(0.511-0.573) | 0.009 |
| SARS-CoV-2 IgM^#^ | 0.606(0.575-0.638) | <0.001 |
| SARS-CoV-2 IgG^*^ | 0.713(0.682-0.743) | <0.001 |
| SARS-CoV-2 IgM^*^ | 0.584(0.552-0.616) | <0.001 |
| WBC* | 0.578(0.547-0.610) | 0.091 |

eGFR: estimated glomerular filtration rate; WBC: white blood cell.

^#^ The result during quarantine observation and rehabilitation training

^*^ The result at the time of diagnosis

**Supplementary Table S3.** Comparison between the area under the curve of the models and each independent risk factor for SARS-CoV-2 re-positivity.

| Comparison | p |
| --- | --- |
| The model vs. the doses of the COVID-19 vaccine | P < 0.0001 |
| The model vs. cumulative frequency of infection with COVID-19 | P < 0.0001 |
| The model vs. eGFR^#^ | P < 0.0001 |
| The model vs. SARS-CoV-2 IgM^#^ | P < 0.0001 |
| The model vs. SARS-CoV-2 IgG^*^ | P < 0.0001 |
| The model vs. SARS-CoV-2 IgM^*^ | P < 0.0001 |
| The model vs. WBC* | P < 0.0001 |

eGFR: estimated glomerular filtration rate; WBC: white blood cell.

^#^ The result during quarantine observation and rehabilitation training

^*^ The result at the time of diagnosis

**Supplementary Table S4.** Demographics and clinical characteristics of the patients in the verification group

| characteristics | verification group **(n=71)** |
| --- | --- |
| Age(year) | 35(29-43) |
| Sex(man) | 56(78.9%) |
| re-positive patients | 31(43.7%) |
| **The doses of the COVID-19 vaccine** |  |
| 0-1 | 6(8.5%) |
| 2-4 | 65(91.5%) |
| **Number of episodes of COVID-19** |  |
| 1 | 66(93.0%) |
| 2 | 5(7.0%) |
| **Laboratory findings** |  |
| White blood cell(10^9/L)* | 6.01(4.94-6.98) |
| eGFR(ml/(min×1.73 m^2^))^#^ |  |
| <90 | 57(80.3%) |
| ≥90 | 14(19.7%) |
| SARS-CoV-2 IgM(S/CO)^#^ | 0.66 (0.18- 5.76) |
| SARS-CoV-2 IgG(S/CO)* | 146.80(52.32- 211.47) |
| SARS-CoV-2 IgM(S/CO)* | 0.60(0.08- 3.69) |


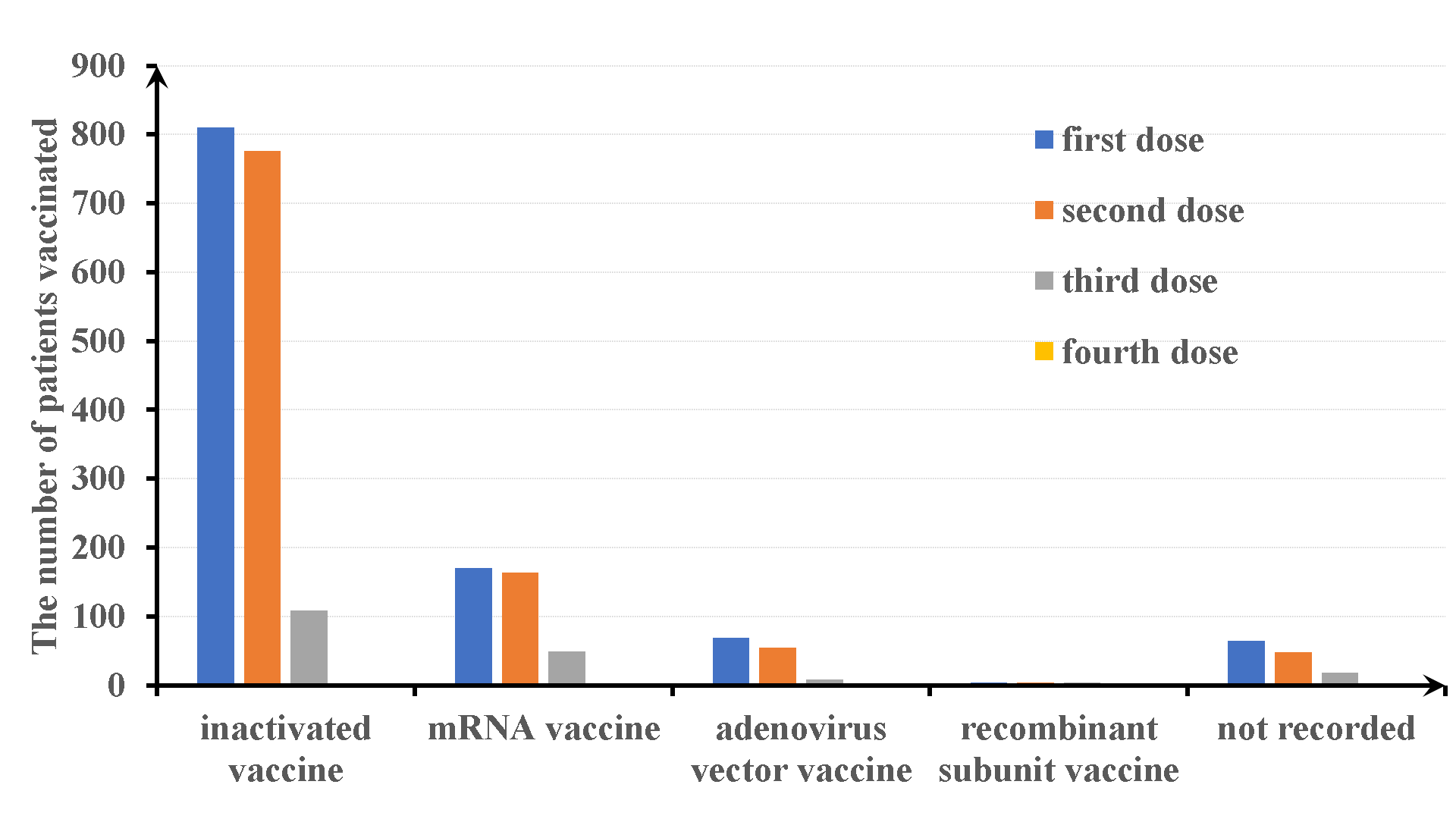
**Supplementary Figure S1.** Vaccination status in the training group


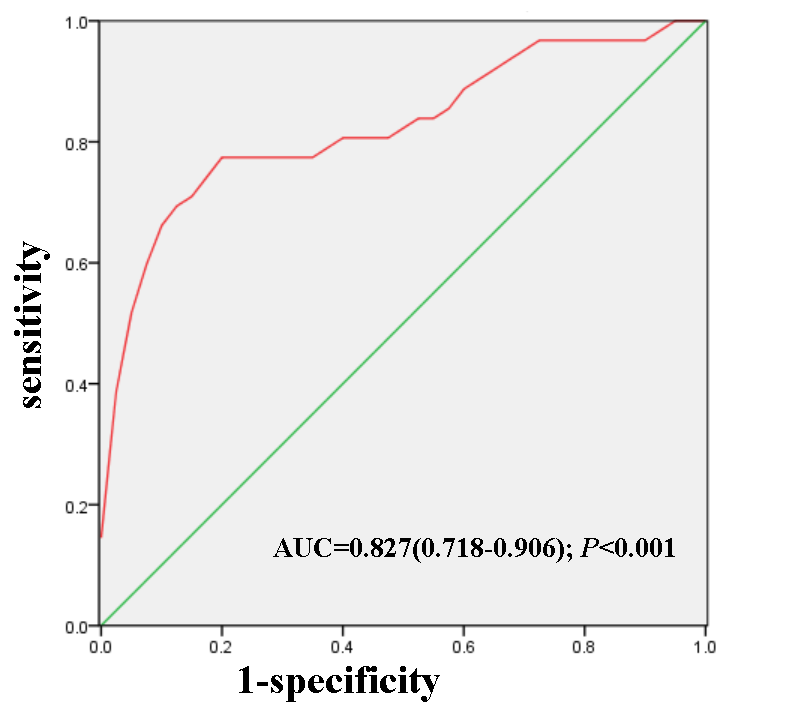
**Supplementary Figure S2.** Area under the curve in the verification group
